# Recurrent Unprovoked Venous Thrombosis (Cerebral Sinus Thrombosis and Mesenteric Vein Thrombosis) in Young Patient with Crohn's Disease: A Case Report and Review

**DOI:** 10.1155/2020/8863900

**Published:** 2020-08-26

**Authors:** Abdullah Mohammed Albishi, Rafaat Chakik, Ali Saleh Alshamrani

**Affiliations:** ^1^Gastroenterology and Endoscopy Department, Armed Forces Hospital Southern Region, Khamis Mushayt, Saudi Arabia; ^2^Internal Medicine Department, Armed Forces Hospital Southern Region, Khamis Mushayt, Saudi Arabia

## Abstract

Inflammatory bowel disease (IBD) patients have a higher risk of thrombosis. Most data about thrombosis in inflammatory bowel disease are related mainly to inpatients with disease activity, but in this article, we report one case of inflammatory bowel disease with two different sites of venous thrombosis which happened in a patient with nonactive IBD at an outpatient setting.

## 1. Introduction

Thrombosis in IBD is associated with a high mortality rate and directly impacts the quality of life of individuals. The literature shows that patients with ulcerative colitis and Crohn's disease have an increased risk of thrombosis, three- to fourfold higher than subjects without inflammatory bowel disease [1].

Deep venous thrombosis of the lower and upper limbs and pulmonary embolism are the most common locations of venous thromboembolism in IBD patients, but unusual sites of thrombosis, including the cerebrovascular, mesenteric, portal, or retinal veins, have also been described [2].

Here we report one case of inflammatory bowel disease with recurrent unprovoked venous thrombosis at two different rare sites which happened in a nonactive patient at an outpatient setting.

## 2. Case Report

A 42-year-old Saudi male patient was diagnosed as colonic inflammatory Crohn's disease 10 years ago on azathioprine. The patient presented to the emergency room (ER) complaining of headache for 2 days which started suddenly and was progressive, generalized, moderate to severe, and not relieved by analgesics. The patient had no history of seizures or decreased level of consciousness, no history of trauma or blurred vision, and no history of weakness, vomiting, fever, and neck pain. He had history of mild headache attacks, but this time it was more severe.

The patient had no history of abdominal pain, diarrhea, skin rash, joint pain, and jaundice. He was taking his medications regularly. He had no history of smoking or using of any NSAIDs and no significant family history. His Crohn's disease activity index (CDAI) was 97.

Regarding Crohn's disease, the patient was diagnosed 10 years ago as inflammatory, nonstricturing, nonfistulazing colonic Crohn's disease without perianal disease when he presented with chronic abdominal pain, chronic diarrhea, and weight loss for more than 3 years before the time of diagnosis. Colonoscopy at the time of diagnosis showed few aphthous colonic ulcers with normal terminal ileum. Colonic biopsy showed chronic colitis. CT enterography was done and showed normal small bowel. The patient was started on steroid therapy for a short period with azathioprine 150 mg orally daily as a maintenance therapy.

Since the time of diagnosis, the patient was following the gastroenterology clinic regularly. He was doing well with no abdominal pain or more diarrhea. He gained weight more than 10 kg within a 4-month period. His routine laboratory investigations were extracted and reviewed which confirm remission of disease clinically and biochemically. The patient was planned for colonoscopy to assess the mucosal and histological remission.

### 2.1. Examination Results

The patient was conscious and oriented to time, place, and person. He was not pale or jaundiced , with normal fundoscopy and normal vital signs and afebrile.

The chest and cardiovascular system were normal.

The abdomen was soft and lax with no tenderness and no organomegaly.

His central nervous system showed normal cranial nerves and motor and sensory and coordination.

No perianal fistula or fissure was seen in the perianal area.

### 2.2. Laboratory Results

CBC, liver function tests, and renal profiles were normal, CRP was 24, and ECG was normal.

At the ER, CT brain was done and was reported as no acute infarction or bleeding or space-occupying lesions.

**Figure 1:**
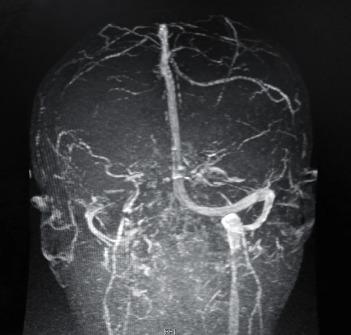
MRI and MRV brain showing no flow within the left transverse and sigmoid sinuses and also no flow within the left internal jugular vein.

MRI and MRV brain showed no flow within the left transverse and sigmoid sinuses and also no flow within the left internal jugular vein as shown in Figure 1.

The patient was diagnosed as cerebral sinus thrombosis, and workup for thrombosis was requested, and the patient started on therapeutic enoxaparin and then on warfarin 5 mg orally daily with target INR 2-3.

ANA, dsDNA, anticardiolipin, lupus anticoagulant, B2 glycoprotein, and JAK2 mutation were all negative. Protein C, protein S, antithrombin III, and peripheral blood smear were all normal.

The patient was following the gastroenterology clinic for 3 years every 6 months and anticoagulation clinic for warfarin adjustment dose.

He was doing well during this period taking azathioprine 150 daily and warfarin 5 mg per day. He was totally asymptomatic. His investigations during the clinic follow-up showed normal CBC, liver function tests, and renal profiles, CRP was 12, and calprotectin was 39. Follow-up MRI and MRV brain which were done after 3 years from the cerebral sinus thrombosis showed no more thrombosis.

The patient was seen by a neurologist, and hematology consultation was requested regarding the possibility of stopping anticoagulant. They labeled him as unprovoked thrombosis with normal thrombosis workup, so anticoagulant was stopped as the multidisciplinary team plan.

**Figure 2:**
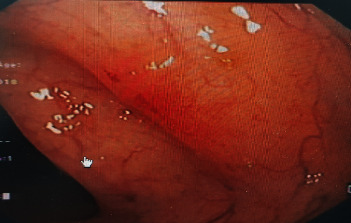
Colonoscopy showed mild patchy erythema at the descending and transverse colon, no ulcers or strictures, and normal terminal ileum.

Colonoscopy was done to assess the remission and showed mild patchy erythema at the descending and transverse colon, no ulcers or strictures, and normal terminal ileum as shown in Figure 2.

After 1 year from stopping anticoagulation, the patient presented again to the ER complaining of abdominal pain for 1 day, which was severe mainly in the epigastrium, sudden onset, associated with vomiting and loose stool, no blood in stool or fever or jaundice, and no urinary symptoms. He was only on azathiopine 150 mg. His CDAI was 149.

### 2.3. Examination Results

The patient was fully conscious, oriented, and in severe pain.

Vital signs were stable apart from mild tachycardia and afebrile. O_2_ saturation was 95%.

His abdomen showed moderate tenderness at the epigastrium, no rebound, and no ascites.

His perianal area showed no perianal fistula and was negative for blood or melena.

### 2.4. Laboratory Results

CBC, liver function tests, and renal profile were normal. Blood gases and lactate level were normal. ECG and cardiac enzymes were normal.

**Figure 3:**
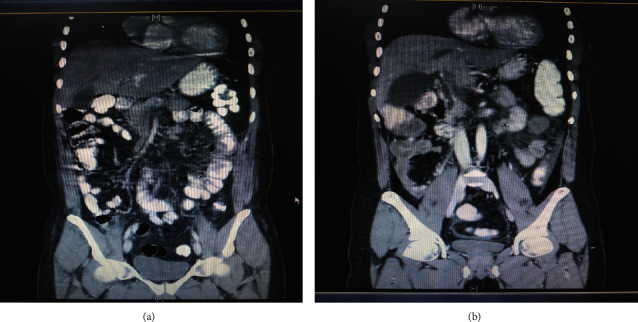
CT abdomen with IV contrast showing mesenteric vein thrombosis and thickening of the small bowel with skipped lesions.

CT abdomen with IV contrast showed mesenteric vein thrombosis and thickening of the small bowel with skipped lesions as shown in Figure 3.

The patient was admitted under the surgery team, and he underwent diagnostic laparotomy because the pain was not relieved by analgesia. No ischemia was found. The patient was discharged on warfarin indefinitely, and he was started on adalimumab.

During clinic follow-up, he was doing well, taking azathioprine 150 daily and adalimumab 40 mg subcutaneously every 2 weeks and warfarin 5 mg per day. He was totally asymptomatic. Investigations during clinic follow-up showed normal CBC, liver function tests, and renal profiles, CRP was 6, and calprotectin was 27.

The patient was instructed to continue the same medications with regular follow-up in the clinic.

## 3. Discussion

Inflammatory bowel diseases (IBD) patients are at high risk of venous thromboembolism (VTE) as a result of the hypercoagulable state induced by chronic inflammation of the bowel [3]. Ongoing active inflammation in IBD itself appears to be a significant factor determining the VTE risk [4].

The most important risk factors for VTE in IBD are active disease, hospitalization, colonic disease, and recent surgery [5]. Around 80% of IBD patients had active disease at the time of VTE diagnosis, which was found in a study done by Dr. Craig A. Solem. Regarding the disease extension in IBD patients with VTE, 79% of Crohn's disease patients had colonic involvement, and 76% of the ulcerative colitis patients had pancolitis as seen in that study [6].

**Figure 4:**
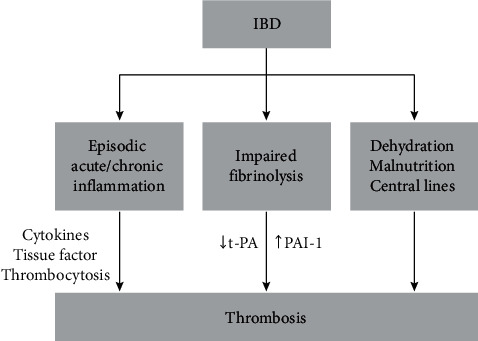
Mechanisms of thrombosis in IBD patients [7].

Acute and chronic episodes of intestinal inflammation lead to the elaboration of inflammatory cytokines and the development of a systemic prothrombotic state, manifested by thrombocytosis, upregulation of tissue factor and impaired fibrinolysis caused by decreased expression of tissue plasminogen activator (t-PA) and increased expression of PAI-1. The recurrent use of corticosteroids for IBD flare may exacerbate the prothrombotic state, leading to further impairment of fibrinolysis as shown in Figure 4 [7].

VTE inherited risk factors occur with a similar frequency in IBD patients and the general population [5].

The VTE overall incidence in IBD patients is estimated to be 1%–8%. IBD patients with VTE episode were generally younger and also associated with a higher risk of recurrence [8].

VTE episode in IBD patients is associated with longer hospitalization and high mortality. Half of the recurrent VTE in IBD happens in patients with active disease at recurrence [5].

Most data about VTE episode in IBD refer to hospitalized active patients. The Canadian Consensus recommends thromboprophylaxis with heparin for hospitalized active IBD flare patients without active non-severe bleeding. For outpatients, thromboprophylaxis is recommended during moderate to severe IBD flares with an unprovoked previous VTE event [2].

There is no evidence for thromboprophylaxis in active IBD patients as outpatients, but the ECCO guidelines recommend that prophylaxis in IBD patients should be considered following discharge from hospital and after recent surgery and in outpatients with active disease [5].

Cerebral sinus thrombosis is rare in IBD patients. Cerebral venous thrombosis is more common in UC than in Crohn's disease [9]. The most common sign is headache, which gradually increases over a period of days or can be sudden severe headache which can mimic subarachnoid hemorrhage [10]. In IBD patients complicated by cerebral venous thrombosis, favorable outcomes are possible with early diagnosis and appropriate treatment; otherwise, the mortality rate can be as high as 50% if not treated [11].

Mesenteric vein thrombosis in IBD patients is difficult to be diagnosed which may delay the diagnosis and initiation of treatment as IBD patients frequently present with nonspecific abdominal discomfort [12].

The current practice in treatment of IBD patients with venous thromboembolism is the same as for non-IBD patients. In the acute setting, anticoagulation with low-molecular-weight heparin or unfractionated heparin is recommended, with transition to oral anticoagulation [13].

**Figure 5:**
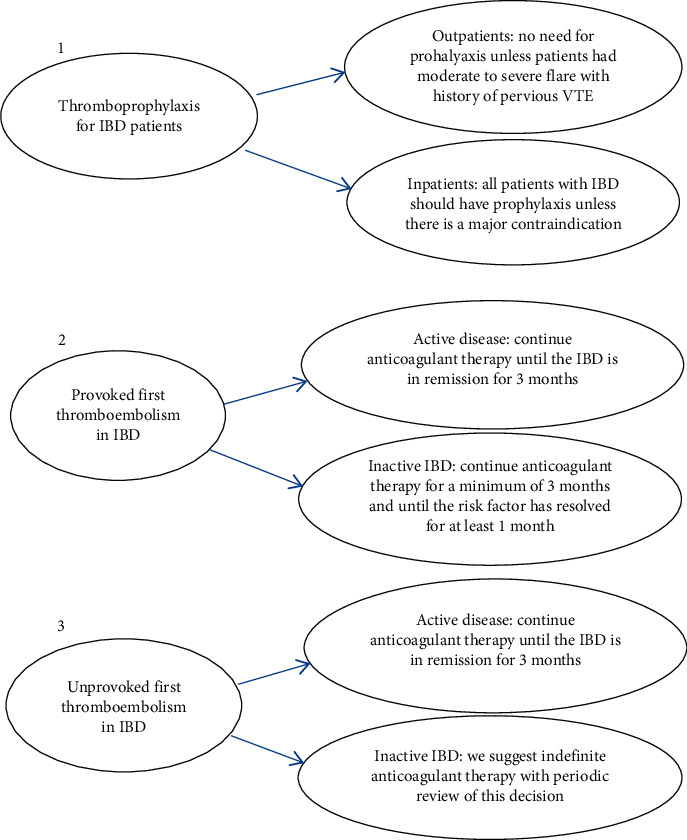
Summary of recommendations of the Canadian Association of Gastroenterology [14].

For patients with IBD at clinical remission who present with provoked venous thromboembolism other than IBD, the duration of anticoagulant therapy is a minimum of 3 months, with continuing therapy until 1 month once the risk factor has been resolved. For IBD patients at clinical remission with unprovoked venous thromboembolism, indefinite anticoagulant therapy is recommended [14]. Recommendations from the Canadian Association of Gastroenterology are summarized in Figure 5 [14].

## 4. Conclusion

Thrombosis either provoked or unprovoked is more reported for patients with IBD.VTE in IBD patients can occur anywhere in the body. High index of suspicion is needed for early diagnosis and appropriate treatment; otherwise, the mortality rate will be high. Management IBD patients at clinical remission with unprovoked thrombosis needs more clarifications.

## Data Availability

The patient was consented, and all data related to the article are available.
